# A Unique Case of Elizabethkingia Associated Cardiac Device Infection

**DOI:** 10.7759/cureus.20028

**Published:** 2021-11-30

**Authors:** Yingyot Arora, Lorenzo D’Angelo-Piaggio, Noah Llaneras, Roger Carrillo

**Affiliations:** 1 School of Medicine, University of Miami Miller School of Medicine, Miami, USA; 2 Cardiology, Palmetto General Hospital, Hialeah, USA; 3 School of Medicine, Herbert Wertheim College of Medicine, Miami, USA

**Keywords:** severe sepsis, pacemaker lead infection, lead complication, lead extractions, cardiac implantable electronic device (cied), elizabethkingia

## Abstract

*Elizabethkingia* is a ubiquitous gram-negative aerobic bacillus that has gained attention in recent years as an emerging nosocomial infection in critically ill patients. We describe a case of bacteremia that developed in a patient who underwent complicated surgery with an extended intensive care unit (ICU) stay. The patient underwent pacemaker extraction with laser lead extraction and treatment with intravenous antibiotics. This case illustrates the importance of lead management strategies in septic patients with cardiac implantable electronic devices (CIED).

## Introduction

Infections are serious and growing complications of cardiac implantable electronic devices (CIEDs). They can occur as a surgical site infection (SSI), typically occurring within a year of implantation, or as late-onset lead endocarditis [1]. Although infections only occur in 1-4% of CIED procedures, they are associated with a three-fold increase in mortality, reduced quality of life, and disruptions in CIED therapy [2]. Treatment involves culture-driven antibiotic therapy and complete removal of all intracardiac hardware, including generator and intracardiac leads. While staphylococcal species are the most common pathogen implicated in infection, common clinical contaminates and otherwise unusual bacterial species should be considered, especially in immunocompromised or frail patients [3]. The patient in this report was infected with the nosocomial Elizabethkingia species, rarely cultured in CIED infections.

## Case presentation

An 86-year-old male with a history of atrial fibrillation and hypertension presented to our emergency department for a three-day history of hematuria with urinary retention and fever. A review of records revealed that a dual-chamber pacemaker was implanted four years ago for the management of chronic atrial fibrillation with symptomatic bradycardia. Cystoscopy suggested a bladder outlet obstruction causing urinary retention, and the patient was scheduled for a radical open prostatectomy. Due to severe anemia (Hemoglobin 8.3 g/dL; reference range 14-18 g/dL), anticoagulants were held, and intervention radiology placed an infrarenal inferior vena cava (IVC) filter before surgery. His prostatectomy was complicated by hemorrhagic shock-receiving six units of packed red blood cells (RBC), platelets, and fresh frozen plasma on the table. Postoperatively, his condition continued to deteriorate, and he was taken back to the operating room the next day. The patient was transferred to the ICU and was extubated. Blood and urine culture revealed pyuria, and the patient was started on vancomycin and cefepime. On postoperative day two, the patient developed labored breathing; bronchoscopy revealed significant thin secretions, and the patient was subsequently reintubated. A follow-up chest x-ray showed bilateral lower lobe parenchymal infiltrates and pulmonary effusions. Blood, urine, and sputum were recultured on postoperative day three. Blood cultures grew *Elizabethkingia,* while sputum cultures grew *Enterobacter*. Antibiogram showed that the microorganisms were sensitive to the levofloxacin and minocycline he was started on.

**Figure 1:**
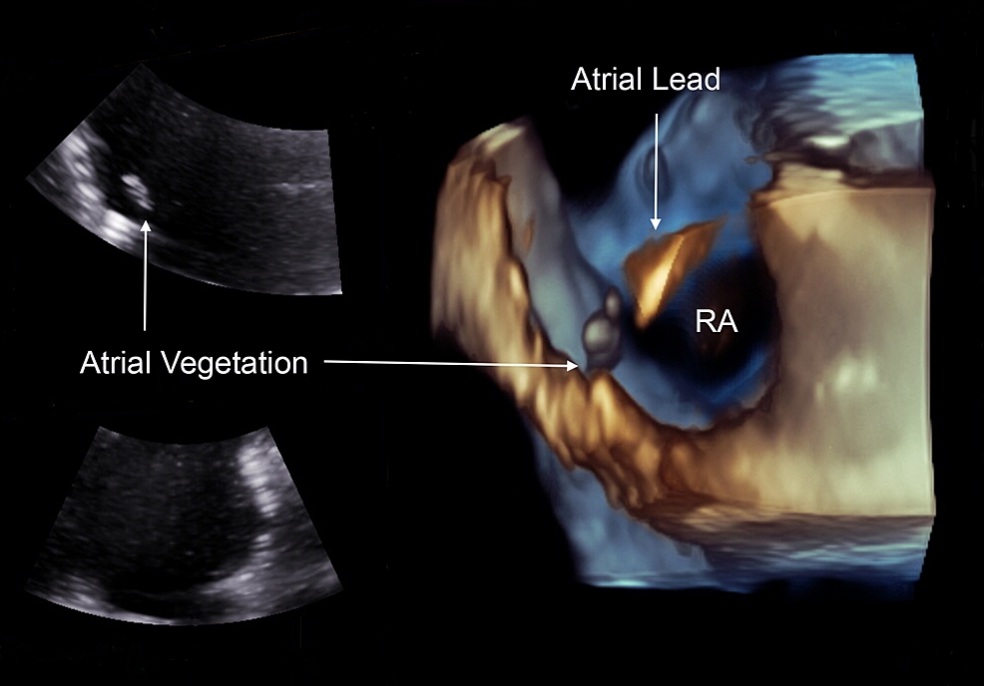
TEE showing a 0.42cm x 1.92cm vegetation attached to the right atrial lead RA - right atrium; TEE - transesophageal echocardiogram

On hospitalization day 10, cardiology was consulted for evaluation of persistent bacteremia. A transesophageal echocardiogram (TEE) revealed an ejection fraction of 55% and a 0.42cm x 1.92cm finger-like vegetation attached to the right atrial lead (Figure 1). He was scheduled for a transvenous lead extraction for removal of all intracardiac hardware the following day.

During the procedure, femoral venous access was obtained through a 12 French sheath. A stiff guidewire was floated to the right internal jugular vein. Significant resistance was encountered at the level of the inferior vena cava. Despite significant manipulation, the catheter could not be advanced past the IVC filter. An endovascular occlusion balloon was placed prophylactically, and the femoral approach was aborted. Instead, an incision was made in the left infraclavicular space along the previous scar for a superior approach. First, the St Jude’s Pacemaker and two leads were dissected in the infraclavicular space, and the active leads were retracted. A lead locking device was placed on the inner channel of the atrial lead. Due to a significant amount of scar tissue, manual traction alone was insufficient to free the lead. Laser techniques were used to release fibrous attachment to the subclavian vein, innominate vein, superior vena cava, and right atrium. Vegetation was attached to the tip of the atrial lead. 

Next, a lead lock was placed on the ventricular lead. Once again, significant adhesions were anchoring the lead to the vessel wall. A laser catheter was used to free the lead from the surrounding intimal tissue, and the lead was removed uneventfully. TEE showed no new changes except for the removal of the leads. The chest x-ray at the end of the procedure was unremarkable. The patient was transferred to the ICU in stable condition. No growth was observed in follow-up cultures of the leads or tissue pocket at 24, 48, or 72 hours.

The patient was weaned from pressors on postoperative day one and was awake and following commands. On postoperative day two, the patient was extubated. On postoperative day four, we implanted a Micra (Medtronic, Fridley, Minnesota) pacemaker. The patient remained on a 42-day course of antibiotics from the date of pacemaker removal. 

## Discussion

*Elizabethkingia*, a ubiquitous gram-negative aerobic bacillus, has gained attention in recent years as an emerging nosocomial infection in critically ill patients. This opportunistic infection, generally found in water and soil, has been associated with high morbidity and mortality rates in critically ill intensive care unit (ICU) patients on hemodialysis. Mortality has been reported to be between 23-41% due to a lack of effective therapeutic regimens and intrinsic antibiotic resistance [4,5]. In our case, a high-risk individual developed sepsis post-prostatectomy. The time from hospital admission to the isolation of the organism was 17 days. This finding is consistent with previous case studies, which have reported that *Elizabethkingia* is associated with late-onset infections, often 5-50 days post hospitalization [6]. Nosocomial colonization of ventilators, endotracheal tubes, and indwelling catheters have been reported as a growing concern as this beta-lactamase-producing species is intrinsically resistant to major antibiotic classes used in the empiric treatment of sepsis. *Elizabethkingia* is resistant to antibiotics targeting gram-negative organisms, including aminoglycosides and polymixins. However, it has been reported to be susceptible to antibiotics used in the treatment of gram-positive infections. Previous case series have reported that a combination regimen of vancomycin and rifampicin provided suitable coverage. However, the efficacy of this treatment has been questioned [7]. In our case, the isolated bacteria was not a polydrug-resistant species. Antibiogram indicated susceptibility to tetracyclines and fluoroquinolones, and a 40-day course of minocycline and levofloxacin was deemed appropriate. 

We conducted a literature review and found no previous reports of *Elizabethkingia* associated cardiac implantable electric device (CIED) vegetations. An analysis of the Multicenter Electrophysiologic Device Cohort (MEDIC) registry suggests that *staphylococcus aureus* is the predominant pathogen in blood cultures of patients with lead-associated endocarditis (LAE). It has also been reported that the incidence of *staphylococcus aureus* and coagulase-negative staphylococci (CoNS) are equal in patients with larger vegetations (>1cm) [8]. Patients who develop LAE within six months of a CIED procedure tend to present with local pocket inflammation and signs of bacteremia. Conversely, patients with late LAE present with fever, chills, and sepsis. As was the case in our patient, late LAE is frequently due to hematogenous seeding of infections from dialysis catheters, central venous catheters, arteriovenous fistulae, or peripheral abscesses. Regardless of infectious etiology, the management of lead-associated endocarditis unequivocally remains the same: prompt evaluation with TEE and subsequent removal of all intracardiac hardware. TEE is the gold standard imaging modality for identifying lead vegetations, as it allows for a thorough evaluation of both vegetation size and morphology. The urgency of early detection cannot be stressed enough. Vegetation size is one of the only modifiable predictors of morbidity and mortality in LAE [9]. Early detection and intervention are imperative to prevent further growth of vegetations and risks associated with removing a device with large vegetations. Delayed extraction (>7 days) has been associated with worse in-hospital morbidity and one-year mortality in patients with bacteremia and isolated pocket infections [10]. Transvenous lead extraction (TLE) has an established safety record when performed by an experienced operator at a high-volume center. The risk of vegetation embolization to the pulmonary arteries remains low, even in cases of very large vegetations (>2cm). Furthermore, catheter-based debulking solutions may be used to significantly reduce vegetation size before proceeding with a standard lead extraction [11]. Even in cases with large vegetations, percutaneous management is generally considered safer than an open chest, on pump approach in a frail patient with multiple comorbidities. 

## Conclusions

CIED infections are associated with significant morbidity and mortality. Atypical presentations of CIED infections, with organisms such as *Elizabethkingia*, can frequently be overlooked in critically ill patients. A high degree of clinical suspicion for LAE is required in chronically intubated patients with CIEDs. Prompt evaluation with TEE, cultures/antibiograms, and percutaneous device removal should be considered in all cases.
